# Leveraging global gene expression patterns to predict expression of unmeasured genes

**DOI:** 10.1186/s12864-015-2250-5

**Published:** 2015-12-15

**Authors:** James Rudd, René A. Zelaya, Eugene Demidenko, Ellen L. Goode, Casey S. Greene, Jennifer A. Doherty

**Affiliations:** Department of Epidemiology, Geisel School of Medicine at Dartmouth College, One Medical Center Drive, 7927 Rubin Building, Lebanon, NH 03756 USA; Department of Genetics, Geisel School of Medicine at Dartmouth College; Department of Systems Pharmacology and Translational Therapeutics, University of Pennsylvania Perelman School of Medicine, 10-131 SCTR, 34th & Civic Center Boulevard, Philadelphia, PA 19104-5158 USA; Department of Biomedical Data Science, Geisel School of Medicine at Dartmouth College, One Medical Center Drive, 7927 Rubin Building, Lebanon, NH 03756 USA; Department of Health Sciences Research, Division of Epidemiology, Mayo Clinic, 200 First St. SW, Rochester, MN 55905 USA

**Keywords:** Gene expression, Greedy gene set selection, GGS, Imputation

## Abstract

**Background:**

Large collections of paraffin-embedded tissue represent a rich resource to test hypotheses based on gene expression patterns; however, measurement of genome-wide expression is cost-prohibitive on a large scale. Using the known expression correlation structure within a given disease type (in this case, high grade serous ovarian cancer; HGSC), we sought to identify reduced sets of directly measured (DM) genes which could accurately predict the expression of a maximized number of unmeasured genes.

**Results:**

We developed a greedy gene set selection (GGS) algorithm which returns a DM set of user specified size based on a specific correlation threshold (|r_P_|) and minimum number of DM genes that must be correlated to an unmeasured gene in order to infer the value of the unmeasured gene (redundancy). We evaluated GGS in the Cancer Genome Atlas (TCGA) HGSC data across 144 combinations of DM size, redundancy (1–3), and |r_P_| (0.60, 0.65, 0.70). Across the parameter sweep, GGS allows on average 9 times more gene expression information to be captured compared to the DM set alone. GGS successfully augments prognostic HGSC gene sets; the addition of 20 GGS selected genes more than doubles the number of genes whose expression is predictable. Moreover, the expression prediction is highly accurate. After training regression models for the predictable gene set using 2/3 of the TCGA data, the average accuracy (ranked correlation of true and predicted values) in the 1/3 testing partition and four independent populations is above 0.65 and approaches 0.8 for conservative parameter sets. We observe similar accuracies in the TCGA HGSC RNA-sequencing data. Specifically, the prediction accuracy increases with increasing redundancy and increasing |r_P_|.

**Conclusions:**

GGS-selected genes, which maximize expression information about unmeasured genes, can be combined with candidate gene sets as a cost effective way to increase the amount of gene expression information obtained in large studies. This method can be applied to any organism, model system, disease, or tissue type for which whole genome gene expression data exists.

**Electronic supplementary material:**

The online version of this article (doi:10.1186/s12864-015-2250-5) contains supplementary material, which is available to authorized users.

## Background

Gene expression studies can reveal genes and pathways critical for specific disease phenotypes [1, 2] and can identify molecular subtypes [3–9], allowing for a better understanding of the etiologies and features of many diseases. The large numbers of formalin-fixed paraffin-embedded (FFPE) tissues which are routinely collected for clinical and diagnostic purposes represent an important resource for genomic studies. While it is possible to perform whole genome expression assays and sequencing in FFPE samples, it is currently cost-prohibitive to do so in the very large collections of FFPE samples that are available. Most FFPE-based research to date has focused on assaying a subset of genes selected based on a current hypothesis of interest (e.g., genes associated with prognosis) or a reduced gene set classifier of molecular subtypes [10–12]. The number of genes included is determined both by scientific rationale and cost, and by definition, represent only a subset of gene expression information. We sought to develop a method to maximize the amount of gene expression information obtained from assayed samples by inferring the expression levels of unmeasured genes.

Conceptually, this problem is similar to genotype imputation. Loci physically located near each other on a chromosome tend to be inherited together, and sets of highly associated loci can be identified using linkage disequilibrium (LD) which is a measure of co-occurrence of alleles. Representative or ‘tag’ single nucleotide polymorphisms (SNPs) from these sets can be selected to be assayed and the remaining values inferred based on LD [13, 14].

In an analogous manner, we propose to use the organism-, disease-, and tissue-specific gene expression correlation structure to identify genes which indirectly provide information about the expression of other genes in that tissue. The correlation of gene expression values is well studied and has been used to help inform molecular pathway definitions [15, 16], disease subtype discovery [3, 7, 8], and clinical prognosis and treatment [5, 6, 17]. Just as it is important to select tag SNPs based on allele correlations in a population similar to the population studied, it is also important to use gene expression patterns from the specific tissue of interest [18]. The robustness of the co-expression relationships directly affects the inference of expression of unmeasured genes; for this reason, our method is valid for stable systems of co-expression, e.g., for design of large-scale targeted assays following initial genome-wide measurements, not dynamic systems such as differentiation where the co-expression relationships are expected to change. We focus on high grade serous ovarian cancer (HGSC) for the development and evaluation of our algorithm, but also apply our method to a breast cancer dataset. The wealth of publicly available expression data allows our method to be used for studies of a wide variety of different organisms, tissues, model systems, and disease types. While our intention is to identify genes that broadly capture gene expression information for many genes, recent work suggests that these genes may also be enriched for disease drivers [19]. Herein, we present our method of gene selection that can be combined with candidate gene sets as a cost-effective way to increase the amount of gene expression information obtained in large studies where using a genome-wide measurement platform is not feasible.

## Results

**Fig. 1:**
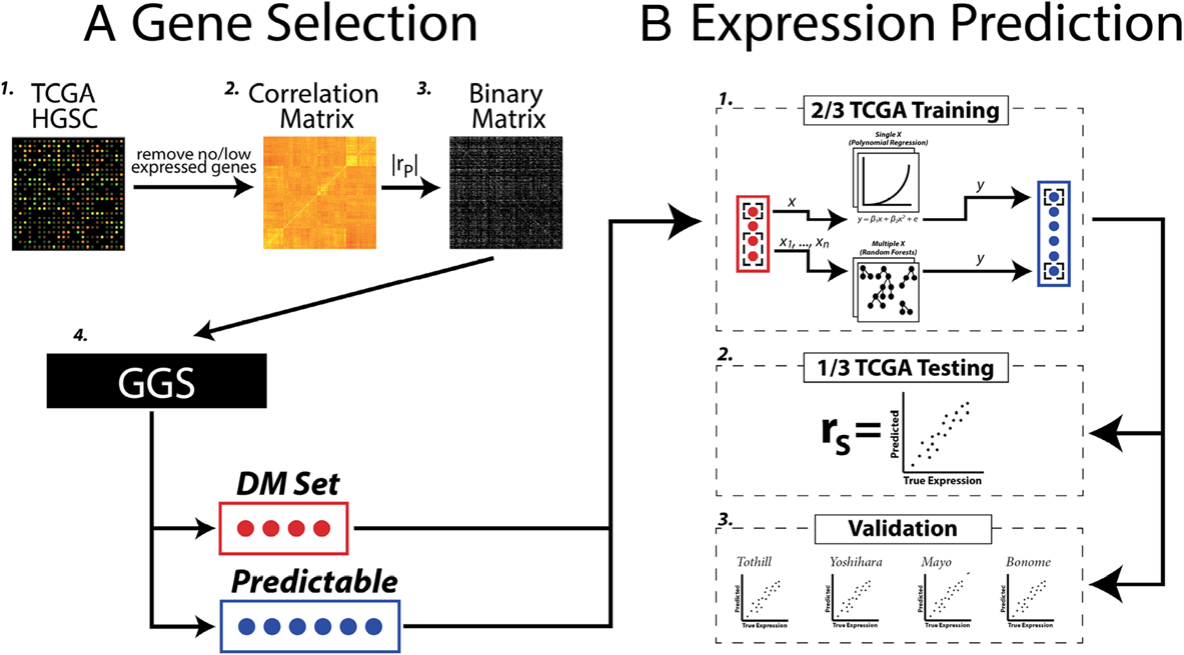
Gene selection **a** and expression prediction **b** workflows. A.1) The workflow starts with the TCGA HGSC Affymetrix gene expression data which is filtered to remove no/low expressed genes. A.2) A symmetrical gene-by-gene correlation matrix is created by calculating pairwise Pearson’s correlation coefficients (r_P_). A.3) A user-determined threshold is applied to the absolute value of the Pearson’s correlation coefficients (|r_P_|) in order to generate a binary adjacency matrix. Here, black indicates no correlation beyond the threshold between two genes, and white indicates the existence of such a correlation. A.4) A greedy geneset selection algorithm iteratively builds a set of genes to directly measure (DM set; red) and a set of genes that are predictable using the DM set (predictable set; blue). The predictable set is defined as those eligible but unmeasured genes that are strongly correlated to at least *n* DM genes where *n* is the redundancy chosen. B.1) The expression prediction workflow starts with splitting the TCGA HGSC data into training and testing partitions. The training partition is used to build a regression model for each gene in the predictable set. Only the genes in the DM set that are correlated to the specific predictable gene above the |r_P_| threshold are used as predictors. If there are multiple predictors, a forest of regression is trained. Otherwise, a polynomial regression of degree 2 is trained. B.2) The regression models are then used in the testing partition of the TCGA HGSC data to predict expression. The true and predicted values are compared using the Spearman rank correlation (r_S_). B.3) The accuracy of the regression models are assessed across populations and platforms using four independent HGSC datasets. In each dataset, the regression models are used to predict expression and r_S_ is calculated

Our greedy geneset selection (GGS) algorithm uses pairwise gene expression correlation (Pearson’s correlation coefficient: r_P_) to identify sets of correlated genes, and within those sets selects genes to directly measure and genes to attempt to infer using the directly measured genes. We applied this algorithm to the Cancer Genome Atlas (TCGA) HGSC data (Affymetrix HGU133a; Fig. 1a), and compared the ability of GGS to maximize the number of inferred genes given a user defined size of directly measured (DM) genes to that of a ranked-degree gene selection method. We tested the ability to infer unmeasured expression by constructing regression models for unmeasured genes and evaluating the accuracy of these predictions in independent studies (Fig. 1b).

### Characterization of eligible genes

**Fig. 2:**
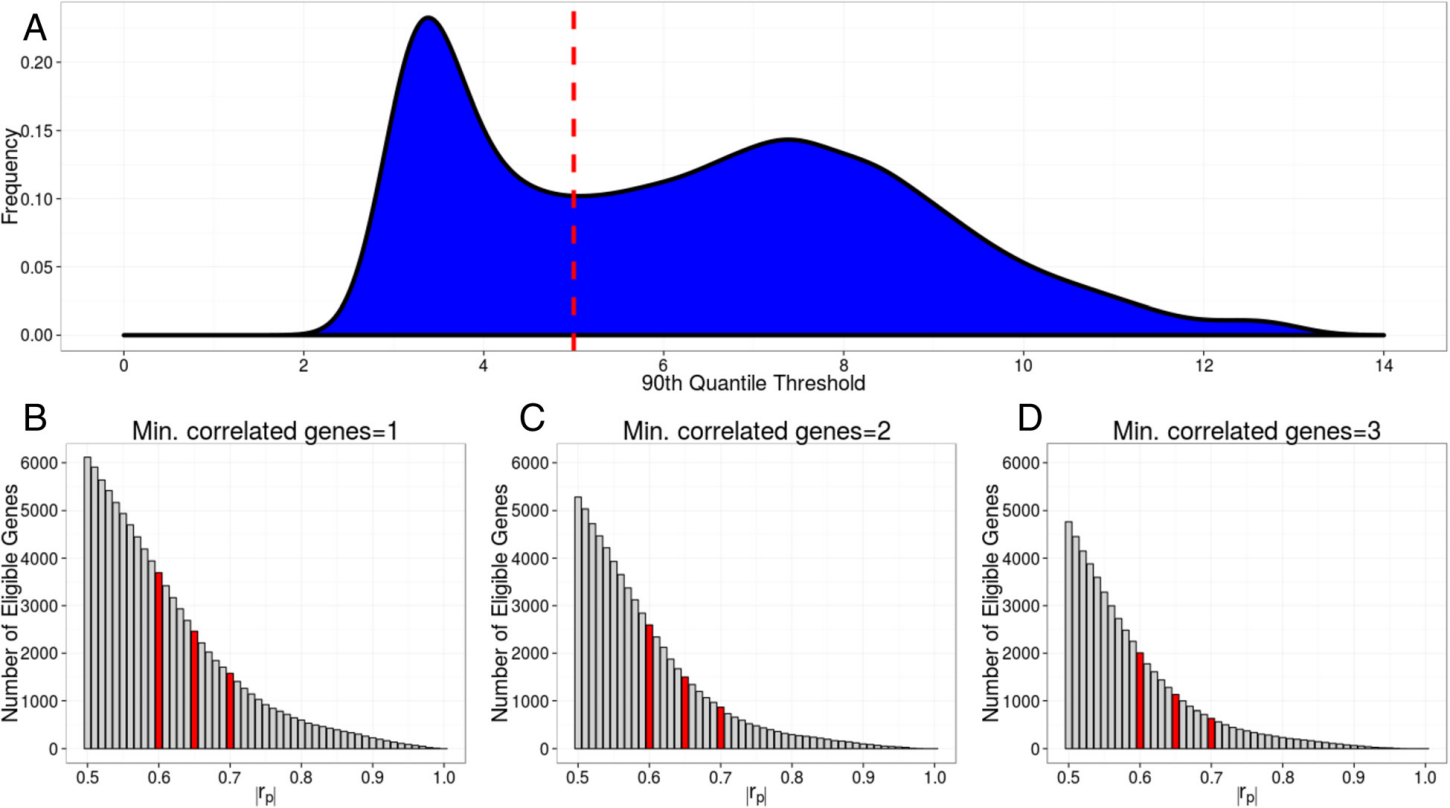
Definition of eligible genes. **a** Distribution of the 90th quantile of expression values for all genes in the TCGA data. The distribution is bimodal, and we excluded genes below the cutoff of 5 (dashed red line) because most samples have low/no expression for these genes. **b** Number of eligible genes (*y-axis*) by the correlation threshold (*x-axis*) when the minimum number of genes to which an eligible gene must be correlated is set to 1. Highlighted in red are specific values when the correlation threshold is set to 0.60, 0.65, and 0.70. **c** Same as B but with the minimum number of genes to which an eligible gene must be correlated is set to 2. **d** Same as B but with the minimum number of genes to which an eligible gene must be correlated is set to 3

We removed genes that did not appear to be expressed (90th quantile thresholds below 5), leaving 8,265 genes for analysis (Fig. 2a). We calculated pairwise correlations between these genes to identify those for which an expression level could be predicted. The minimum number of genes to which an eligible gene must be correlated in order for it to be eligible was set at 1, 2, or 3, for three different correlation thresholds (|r_P_|; absolute value of the Pearson’s correlation coefficient) of 0.60, 0.65, or 0.70. GGS selects the directly measured genes and the theoretically predictable genes from these eligible genes. The number of genes eligible to be directly measured or predicted varies as a function of the correlation threshold and the minimum number of genes to which an eligible gene must be correlated (Fig. 2b–d). More stringent correlation thresholds reduce the number of genes correlated to at least one other gene at or above that threshold. When the minimum number of correlated genes is 1 and |r_P_| is 0.60, 0.65, and 0.70, there are 3,695, 2,463, and 1,577 eligible genes (i.e., genes that can be used to predict or genes whose value can be predicted) respectively. To determine the extent to which the eligible genes represent a wide variety of biological processes, we performed enrichment analysis on the Protein Analysis Through Evolutionary Relationships (PANTHER) GO-slim biological process terms (223 terms) using the 3,695 eligible genes identified using the 0.60 threshold with background frequencies determined by the 8,265 truly expressed genes. After applying a Bonferroni adjustment for the 223 enrichment tests, only 8 processes were underrepresented, and 4 processes were overrepresented at a *p*-value cutoff of *p* < 0.05 (translation; nucleobase-containing compound metabolic process; protein metabolic process; regulation of translation; Additional file 1: Table S1). This suggests that the distribution of the eligible genes may be generally representative of the distribution of all expressed genes across most of these high-level biological processes.

### GGS-selected gene sets can predict the expression of a larger number of genes compared to ranked-degree-selected gene sets

**Fig. 3:**
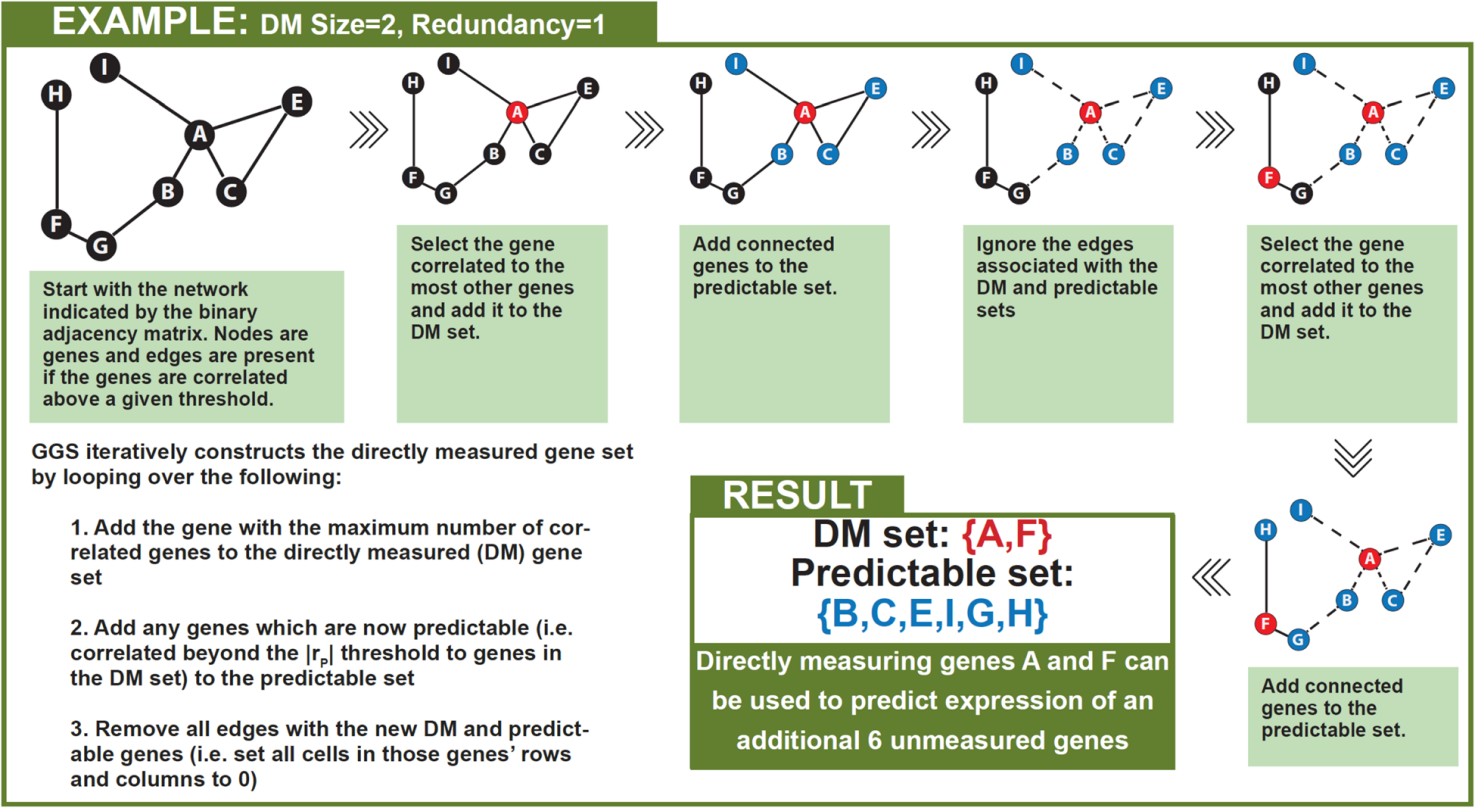
Example run of greedy geneset selection (GGS). We represent the binary adjacency matrix as a network in which the nodes are genes, and an edge exists if the genes are correlated beyond the |r_P_| threshold. DM size and redundancy are set to 2 and 1 respectively

We compared the performance of GGS to a ranked-degree method using all TCGA samples. The ranked-degree method builds a set of *n* genes to directly measure by selecting the top *n* genes correlated with the largest number of genes. In contrast, as GGS constructs the DM and predictable sets, the edges are removed; i.e., correlations associated with those genes are ignored for the remainder of the set construction (Fig. 3). Both approaches require: 1. a binary matrix indicating whether pairs of genes are correlated beyond a specified threshold (|r_P_|); 2. a minimum number of directly measured genes which must be correlated with an unmeasured gene in order to consider that unmeasured gene predictable (redundancy); and 3. the targeted size of the DM set. We used the TCGA HGSC gene by gene correlation matrix and three |r_P_| values (0.60, 0.65, 0.70) to create binary matrices, then applied both the ranked-degree and GGS approaches specifying redundancy as 1, 2, or 3 and a targeted DM set size of 400, and calculated the size of the resulting predictable sets (Table 1). GGS consistently returned at least approximately three times the number of predictable genes across this range of redundancy values and |r_P_| thresholds. Under the most conservative parameters, with redundancy of 3 and |r_P_| = 0.70, the GGS predictable set was approximately 11-fold larger than that of the ranked-degree approach. Therefore, the edge removal portion of the algorithm likely improves GGS performance by preventing over-representation of correlated genes in the DM set.

**Table 1:** Number of predictable genes using GGS versus a ranked-degree approach, by correlation threshold and redundancy

|r_P_|	Redundancy					
	1		2		3	
	Ranked-Degree	GGS	Ranked-Degree	GGS	Ranked-Degree	GGS
0.60	439	2219	355	1200	294	849
0.65	235	1467	170	745	136	518
0.70	102	1018	53	466	25	337

### Predictable gene set size across GGS parameter values and for candidate gene sets

**Fig. 4:**
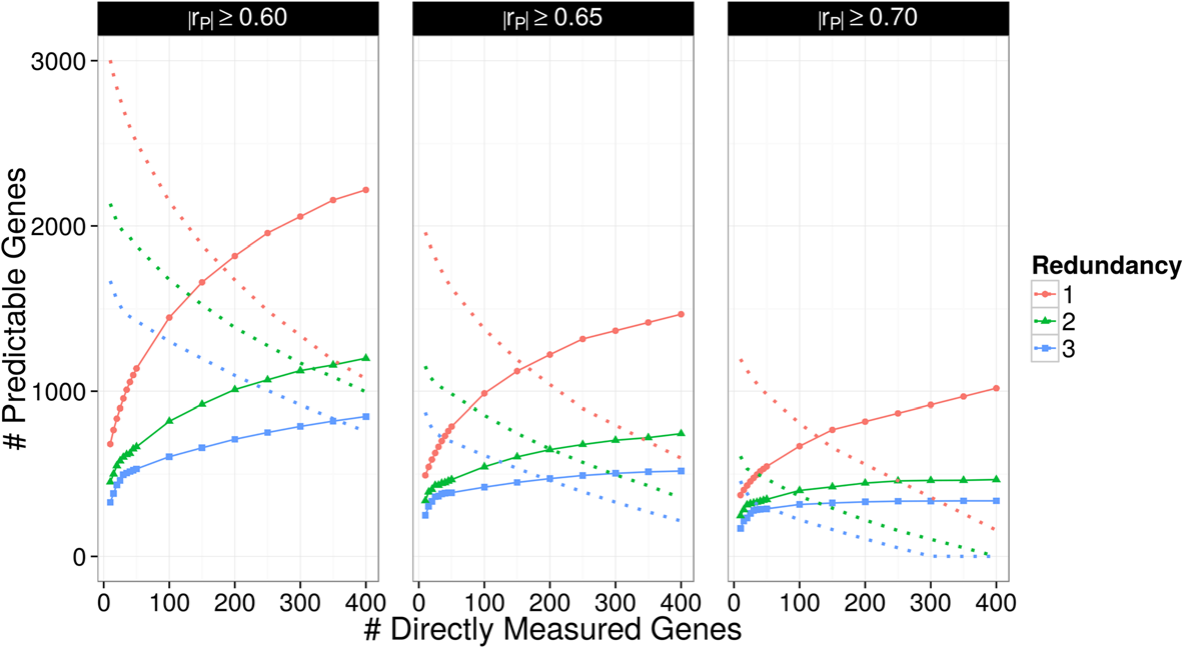
Predictable gene set size across GGS parameters. The number of predictable genes by fold redundancy (1-*red circle*; 2-*green triangle*; 3-*blue squares*), number of directly measured genes (*x-axis*), and correlation threshold (|r_P_|, column facets). Solid lines indicate the number of predictable genes given a GGS-selected DM set of size indicated by the x-axis. The dotted line indicates the remaining eligible genes that are neither predictable nor directly measured

We performed a parameter sweep across |r_P_| of 0.60, 0.65, and 0.70, redundancy of 1, 2 and 3, and DM set size (16 values between 10 and 400), totaling 144 individual GGS runs with resulting DM sets. For each of the DM sets, the size of the corresponding predictable set was calculated (Fig. 4). As shown in Fig. 2b–d, the total number of eligible genes for given parameter values is known, and we subtracted the number of genes in the DM and predictable sets from the number of eligible genes to quantify the eligible genes missed (dotted lines in Fig. 4). A consistent pattern was observed; the size of the predictable set increased with increasing DM set size and decreasing |r_P_| and redundancy. Redundancy strongly influences the number of predictable genes. For example, for |r_P_| of 0.60 and a DM set size of 250, the number of predictable genes is 2.6-fold higher when redundancy is 1 (1,954 genes) compared to redundancy of 3 (752 genes). The correlation threshold (|r_P_|) also has a strong effect; when redundancy is 1 and the DM set size is 250, the number of predictable genes is 2.3-fold higher for |r_P_| of 0.60 (1,954 genes) compared to |r_P_| of 0.70 (868 genes). The increase in number of predictable genes as the number of directly measured genes increases is expected. However, a plateau is reached as the size of the DM set increases. This plateau is caused by GGS exhausting the larger sets of correlated genes, and subsequently adding genes to the DM set with a smaller return in increased predictable set size. A network representation of the eligible genes when |r_P_| is 0.70 and redundancy is 1 is presented in Additional file 2: Figures S2 and S3. The average number of neighbors was approximately 9. The GGS-identified DM set genes are red (20 genes Additional file 2: Figure S1; 400 genes Additional file 2: Figure S2), the predictable genes are blue (430 genes Additional file 2: Figure S1; 1018 genes Additional file 2: Figure S2), and the remaining eligible genes are grey. GGS selects from the dense neighborhoods first but with 400 genes in the DM set the algorithm has started to select from the small 2 node connected components which means only 1 predictable gene is gained for every DM gene added. This explains the diminishing returns in number of predicted genes observed in Fig. 4 which occurs when selecting from the small connected components.

We performed parallel analyses of breast cancer gene expression data from TCGA. We observed results similar to the HGSC datasets, with increasing predictable set size as a function of increasing DM size, decreasing redundancy, and decreasing correlation threshold. Predictable set sizes were consistently larger than those found in HGSC. For example, with a set of 400 DM genes and a correlation threshold and redundancy of 0.70 and 3 respectively, 1,566 predictable genes were identified in the breast cancer data, versus 337 predictable genes observed in the HGSC data. These results are provided in our source code repository [20].

We developed GGS to augment hypothesis-driven candidate gene sets with small numbers of additionally measured genes that allow inference of many unmeasured genes. In this scenario, GGS automatically adds all of the candidate genes to the DM set and selects additional DM set genes using the specified binary adjacency matrix. To characterize the performance of GGS with candidate gene sets, we performed the parameter sweep using either the Yoshihara et al. [21] or TCGA [9] prognostic gene sets for HGSC which contain 121 and 183 genes respectively. Starting with a DM set that includes the candidate gene set, the number of predictable genes was quantified across the remaining parameter sweep categories (Additional file 2: Figures S4). The Yoshihara and TCGA candidate gene sets predicted 572 and 224 genes respectively when |r_P_| was 0.60 and redundancy was 1. These candidate gene sets were created to capture specific biological signals and are not optimized to predict unmeasured gene expression (in comparison, a GGS-generated DM set of 100 genes returned by GGS predicted 1447 genes). Once GGS augmented the Yoshihara et al. [21] and TCGA [9] candidate gene sets with 20 additional DM genes, they predicted 968 and 935 genes respectively. This suggests that with a minimal investment in additional assayed genes, GGS can more than double the amount of gene expression data captured.

### Using directly measured genes as predictors, regression models predict unmeasured expression values with high accuracy

To test whether the DM set accurately predicted unmeasured genes, we built a regression model for each gene in the predictable set using the TCGA training partition (2/3 of data) (Fig. 1b). For a specific predictable gene, only the genes in the DM set that were correlated beyond the |r_P_| threshold were used as predictors in the regression model. To evaluate the performance of the regression models, we predicted expression of specific genes using the regression models in the TCGA testing partition (other 1/3 of data), and then correlated the true and predicted values using the Spearman rank correlation (r_S_). Expression prediction was carried out for all parameter sets defined by the parameter sweep (144 GGS runs) and for the parameter sweep results with the two candidate gene sets.

**Fig. 5:**
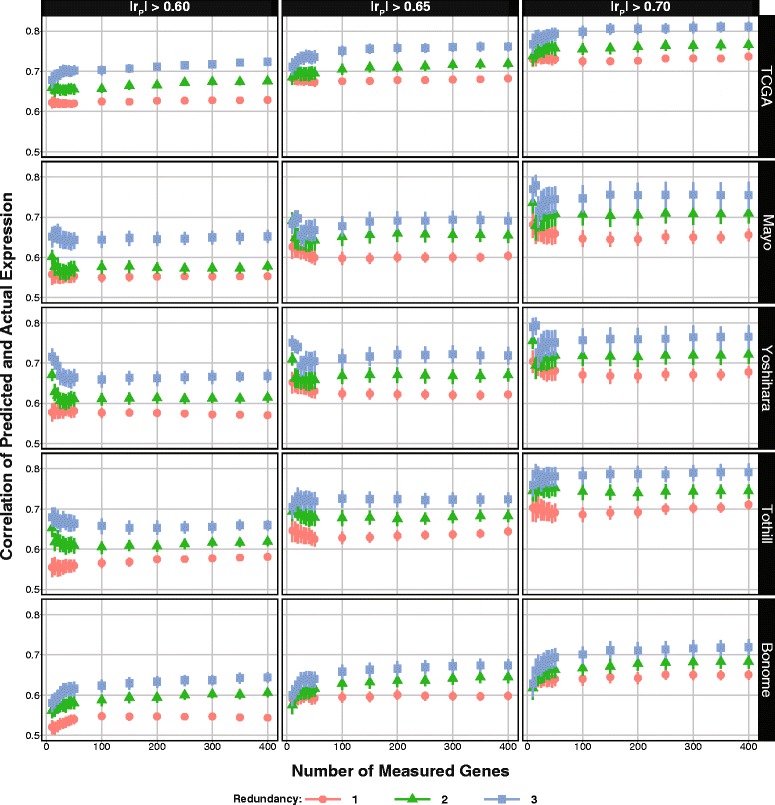
Expression imputation accuracy. Average expression prediction accuracy by number of directly measured genes (*x-axis*), fold redundancy (*color*), correlation threshold (*columns*), and dataset (*rows*). The y-axis indicates the mean and bootstrapped standard error of the Spearman rank correlation between actual expression and predicted expression

We also assessed the performance of these regression models in four independent HGSC expression datasets (Tothill [8], Mayo, Yoshihara [21], and Bonome [22]) (Additional file 3: Table S2, Fig. 1B). We summarized the accuracy (r_S_) of the regression models in the TCGA testing partition and the four independent datasets (Fig. 5), and repeated analyses including the two candidate gene sets (Additional file 2: Figure S4 and S5). We observe similar average r_S_ across most datasets for a given set of parameters. As expected, the average accuracy of our prediction increases as redundancy and |r_P_| increase. In most of the data sets (TCGA [9], Tothill [8], and Bonome [22]), accuracy generally increases with increasing DM set size. However, in the Yoshihara and Mayo data, the maximum r_S_ is achieved when the DM set size is very small (10–20 genes); as more genes are predicted the average r_S_ slightly decreases and levels out (e.g., 3-fold redundancy with |r_P_| = 0.70 in the Yoshihara and Mayo datasets). A similar pattern is observed when using GGS augmented candidate gene sets across all datasets (Additional file 2: Figure S4 and S5). The highest r_S_ is achieved using the candidate genes alone to predict a relatively small number of genes. However, as the DM set size increases and there is a concomitant increase in the number of predicted genes, the r_S_ decreases and levels out. Importantly, when redundancy is 3-fold the average r_S_ (i.e., imputation accuracy) consistently exceeds the |r_P_|used to identify the genes in the DM and predictable sets (in all data except for Bonome et al.; Fig. 5). The highest confidence in imputation accuracy is achieved with redundancy of 3 and |r_P_| 0.70.

For 236 of the TCGA samples assayed on the Affymetrix platform, RNA sequencing (RNA-seq) gene expression data is also available. We used the regression models to predict expression in these samples and in the subset of these samples, which were included only in the TCGA testing partition (*n* = 91; Additional file 2: Figure S6). The overall pattern of r_S_ is similar to that observed in the validation datasets.

## Discussion

Large collections of paraffin-embedded tissue are a rich resource to test hypotheses based on gene expression patterns; however, measurement of genome-wide expression is cost-prohibitive on a large scale. Using the known expression correlation structure within HGSC, we demonstrate that our GGS approach can efficiently identify reduced sets of directly measured genes which accurately predict a maximized number of unmeasured genes in independent data sets, with the ranked correlation between true and predicted expression of 0.60 or greater in all testing scenarios, and nearing 0.80 for conservative parameters. This testing accuracy was observed across Affymetrix and Agilent mRNA expression array platforms and was also demonstrated in RNA-seq expression data. While we emphasize the utility of GGS for the selection of genes to be assayed in future studies, it can also be used to increase the utility of existing targeted gene expression data by using the existing gene set to impute predictable genes.

Gene expression covariance relationships are highly tissue-specific [23], and successful GGS-based expression prediction requires a stable tissue-specific co-expression structure. We demonstrate that in two very different cancer types, breast and HGSC, the same trends in predictable gene set size as a function of redundancy, correlation threshold, and DM set size were observed. The number of predictable genes was consistently much higher in breast cancer than in HGSC. When predicting gene expression, increasing the number of genes required to be correlated with a predictable gene tends to increase the prediction accuracy, since more predictor variables are added to the regression models. However, it also decreases the total number of eligible genes that could either be predicted or used for the prediction. The overall success of expression prediction depends on the redundancy and |r_p_| selected and may vary by tissue type. If the tissue-specific gene expression correlation structure has fewer but larger sets of correlated genes, then larger redundancy values will have little impact on the number of eligible genes. If, however, there are many small sets of correlated genes, high redundancy values would exclude many of the genes from being eligible. In selecting gene sets to assay, higher values for redundancy may be chosen to better accommodate probe failures, but such failures will result in decreased accuracy.

There are several key differences between our work and the National Institutes of Health Library of Integrated Cellular Signatures (LINCS) program selection of a set of 1000 “landmark” genes that can be used to infer 80 % of the genome. The goal of the LINCS project is to increase the capacity of high throughput screening and generation of expression signatures for small molecules across cell lines. The 1000 genes were purposely selected based on their minimally correlated expression across a large number of cell lines, and their utility in inferring the expression of other genes [24]. In contrast, we designed GGS to tailor gene selection using organism-, disease-, and tissue-specific gene expression patterns, identify genes that can be imputed from a given candidate gene set, and select a user-specified number of additional genes to assay which maximize the gene expression information obtained. Additionally, we use a range of correlation thresholds and redundancy to identify gene sets whose values can be imputed with varying degrees of confidence, allowing the user to choose a set of parameters that balances cost and prediction accuracy.

While we demonstrate that GGS-augmented prognostic ovarian cancer gene sets greatly increase the number of genes that could theoretically be predicted, and the prediction models using these genes generalize across studies and platforms, GGS has several limitations. In order to apply our algorithm, a binary correlation matrix must be generated using readily available expression data, which may not exist for a given tissue type or disease. Also, while it is possible to consider higher order interaction between gene expression values, we simplify our method by only considering pairwise correlation between genes. Despite this simplistic modeling of co-expression relationships, we achieve high imputation accuracy across populations and platforms. Another possible limitation is that DM gene set performance can suffer from population or study variance in the correlational structure. For example, imputation accuracy is lower in the Bonome et al. data compared to the other data sets we evaluated, suggesting that the correlational relationships differ between these populations. Since there are various methods to define grade [25] and there have been changes over time in the groupings of histologic types of ovarian cancer [26], this could potentially be due to differences in the characteristics of cases included in the studies. Finally, our choice of a greedy algorithm balances the need for DM sets that maximize the number of predictable genes while minimizing the running time, and therefore there is no guarantee that the DM set selected is optimal. A brute force approach which would guarantee the optimal DM set selection that truly maximizes the possible predicted genes would increase the running time by many orders of magnitude. In contrast, our greedy algorithm runs with the number of iterations equal to the number of DM genes the user desires. While a variety of methods could have been used to predict relative expression, we chose polynomial regression and random forest models because of their simplicity. Predicting relative expression is useful for associative analyses of subtype, outcome, or other sample features, and is more resilient to differences in batch, platform, and population than predicting absolute expression. If absolute expression is modeled and predicted, care should be taken to address these issues.

In summary, we demonstrate that GGS augments candidate gene sets selected for their biologic relevance by increasing the amount of gene expression information captured from the assay and potentially providing preliminary support for future work.

## Conclusions

For a given tissue, disease, organism, or model system, GGS can select a set of genes to directly measure that efficiently capture the expression levels of additional genes across populations and assay platforms. GGS can build from candidate gene sets as a cost-effective way to increase the amount of gene expression information obtained in very large studies where using a genome-wide measurement platform is not feasible. This improves the utility of existing studies and enhances the efficiency of future studies by allowing researchers to use both the directly measured and predicted expression values to test unknown and difficult to anticipate future hypotheses.

## Methods

### Datasets and sample inclusion criteria

All data used for these analyses were de-identified and publicly available. Data were primarily obtained from the R package curatedOvarianData version 1.3.4 [27]. We included only non-custom mRNA array datasets containing at least 100 HGSC or high grade endometrioid tumors with data on at least 10,000 genes: The Cancer Genome Atlas (TCGA [9]; *n* = 549; Affymetrix human genome U133a); TCGA [9] (*n* = 236; Illumina HiSeq RNA sequencing); Yoshihara et al. [21] (*n* = 260; GSE32062; Agilent whole human genome microarray 4x44k); Tothill et al. [8] (*n* = 242; GSE9891; Affymetrix human genome U133 Plus 2.0); and Bonome et al. [22] (*n* = 185; GSE26712 Affymetrix human genome U133a). We also included data published by Konecny et al. [28] consisting of 174 HGSC samples and additional unpublished data from the same group (total *n* = 379; GSE74357 Agilent whole human genome microarray 4x44k, termed Mayo in this manuscript) (Additional file 4: Table S3). These samples were collected under a protocol approved by the Mayo Clinic Institutional Review Board.

Expression data and other variables for all samples were compared within and between datasets using the R package doppelgangR (https://github.com/lwaldron/doppelgangR) which correlates sample pairs of expression vectors and transforms the correlation coefficients using the arc tangent hyperbolic function so that outliers (significantly similar or dissimilar sample pairs) can be identified. Sample pairs with significantly similar expression vectors were marked as duplicates and dropped. If a pair was significantly similar in both expression and other variables (e.g., age, grade, stage, survival, etc.), one member of the pair was kept.

Distinct datasets were chosen for discovery of the DM and predictable sets using GGS, and validation of expression prediction for GGS-selected DM sets. We used the Affymetrix data from TCGA [9] (*n* = 549; genes = 13,104) as a discovery data set. We also used these data to build and evaluate the expression prediction regression models, divided into training (2/3, *n* = 366) and testing (1/3, *n* = 183) partitions. We then assessed the performance of the predicted expression regression models in four independent datasets: Mayo; Yoshihara et al. [18]; Tothill et al. [8]; and Bonome et al. [22], as well as in the TCGA RNA sequencing data. Analyses were also performed using TCGA breast cancer RNAseq expression data [29] provided in the Firehose data repository and accessed using the R package “RTCGAToolbox” [30]. All breast cancer samples available from the TCGA firehose repository (dated 2015-04-02) were used.

### Definition of eligible genes

Analyses were restricted to genes that were expressed. To determine which genes to include, we examined the distribution of each of the 13,104 gene’s 90th quantile threshold of expression in the full TCGA data set (549 samples) (Fig. 2a). The distribution is bi-modal and similar bi-modal distributions were found using the 95th and 99th quantile (data not shown). We chose the value 5 as a threshold as it consistently falls between the two distributions when using the 90th, 95th, and the 99th quantiles; values above 5 were considered truly expressed whereas those below 5 expressed at a low level or not at all. Analyses are restricted to those genes above the threshold (8,265 genes). We next count the number of genes correlated to at least one, two, or three other genes at the |0.60|, |0.65|, and |0.70| Pearson’s correlation (|r_P_|) thresholds (Fig. 2b–d). We define these genes as the eligible gene sets; these genes can be selected by our method as either directly measured genes or predictable genes. A network representation of the eligible genes when |r_P_| is 0.70 and redundancy is 1 was generated using Cytoscape 3.2.1 using a prefuse force-directed layout (Additional file 2: Figures S2 and S3).

### Characterization of genes eligible for DM or predictable sets

Using the TCGA discovery data, we identified all genes that were eligible to be included in either the DM or predictable set when |r_P_| is 0.60 and redundancy is 1; i.e., all genes that are correlated with r_P_ > = 0.60 or r_P_ < = −0.60 to at least 1 other gene. Using the PANTHER human GO-slim [31, 32] biological process pathways (223 terms), we tested the distribution of the eligible genes across pathways in comparison to the 8265 truly expressed genes. The binomial test *p*-values were Bonferroni adjusted to account for multiple testing.

### Selection of genes to directly measure and genes to predict

We implemented a greedy algorithm to select the DM gene set that provides the most information about unmeasured genes and compared it to a ranked-degree approach as a control. Both approaches take as input parameters: 1) a binary adjacency matrix which indicates which pairs of genes are correlated above a certain threshold, 2) the number of genes the user wishes to directly measure, and 3) the fold redundancy. The binary adjacency matrix is created by applying a user selected correlation threshold (|r_P_|) to the gene by gene correlation matrix. Cells in the adjacency matrix are 1 if the two genes are correlated above the threshold and 0 otherwise. Redundancy is defined as the number of genes in the DM set that must be correlated with an unmeasured gene above the |r_P_| threshold in order to consider that unmeasured gene to be predictable. The ranked-degree approach sorts the genes by the number of genes that they are correlated with at a specified threshold, then selects the first *n* highest rank genes where *n* is the desired number of directly measured genes. In contrast, GGS iteratively constructs the DM gene set by looping over the following four key operations (Fig. 3) until the DM set reaches the specified size:

1. Sort the genes in the binary matrix by the number of genes that they are correlated with at a specified threshold
2. Add the gene with the maximum number of correlated genes to the DM gene set
3. Add any genes which are now predictable (i.e., correlated beyond the |r_P_| threshold to the redundancy number of genes in the DM set) to the predictable set
4. Remove all edges with the new DM and predictable genes (i.e., set all cells in those genes’ rows and columns to 0)

Given an undirected gene co-expression graph G = (V,E) for which |V| = *n* and a user specified *k* where |DM| = *k*, the time complexity required to sort the nodes by their degree is O(*n* log(*n*)) and the time required to remove edges is bound by O(*n*^2^) resulting in an overall time complexity of O(*kn*^2^). Additionally, the co-expression graph is stored as an adjacency matrix making the space complexity O(*n*^2^).

The GGS analysis returns the DM set along with the final subset of predictable genes (i.e., eligible but unmeasured genes that are correlated at the specified threshold to at least the specified redundancy number of genes in the DM set). If candidate genes are provided, GGS automatically adds all of them to the DM set and proceeds as described above. We examined the performance of GGS with two candidate gene sets developed to predict survival published by Yoshihara et al. [21] (126 genes) and TCGA [9] (200 genes). These two gene sets were chosen because they have been shown to be most predictive compared to other published survival signatures [17]. After mapping the candidate gene lists to standardized gene symbols using the R package HGNChelper and restricting to the intersection with the curatedOvarianData expressionSet, 121 and 183 genes were left for analysis from the Yoshihara et al. [21] and TCGA [9] gene lists respectively.

While holding the size of the desired DM set constant at 400, we compared the size of the predictable set returned by the ranked-degree and GGS methods when redundancy was set to 1, 2, or 3 and |r_P_| was set to 0.60, 0.65, or 0.70. These |r_P_| cutoffs correspond to r^2^ values for which one member of the gene-by-gene pair explains 36, 42, or 50 % of the variance of the other respectively. We further assessed the performance of GGS by performing a parameter sweep across DM set sizes of 10, 15, 20, 25, 30, 35, 40, 45, 50, 100, 150, 200, 250, 300, 350, and 400, for a total of 144 GGS runs. Parallel analyses were also performed on the TCGA breast cancer data.

### Expression prediction

Once GGS identified the DM and predictable gene sets, for each predictable gene, we tested how well the DM set inferred unmeasured expression. First, all gene expression vectors were scaled to the range [0,1]. Using 2/3 of the TCGA Affymetrix data (*n* = 366) as a training partition, we built a regression model for each gene in the predictable set. Genes in the DM set that were correlated with the specific predictable gene above the specific value of |r_P_| were used as predictors, and when there were at least 2 predictors for a predictable gene, a random forest of regression trees (R package randomForest [33]) was used to generate the model. Otherwise, a polynomial regression model with degree 3 was used (both *x* and *x*^*2*^ terms were included). In the remaining 1/3 of the TCGA data (*n* = 183) we predicted expression using the regression models and then correlated the true and predicted values using the Spearman rank correlation (r_S_). To summarize prediction accuracy across all predictable genes defined by a specific parameter set, we report average r_S_ and a bootstrapped standard error. Only regression models for which the response variable and all predictors were present in the dataset were used. Expression prediction was evaluated for all combinations of |r_P_|, redundancy, and DM set size. For each of the 144 parameter combinations, the predictable gene set was determined and regression models were trained in the TCGA training partition and tested in the TCGA testing partition (analogous analyses were performed using the TCGA breast cancer data). We tested the regression models in the additional four independent HGSC datasets (Mayo, Yoshihara et al. [21], Tothill et al. [8], and Bonome et al. [22]). To assess how well the regression models performed in non-array expression data, we determined average r_S_ for the 236 samples for which TCGA provides both Affymetrix and RNA-seq expression values; we similarly calculated average r_S_ for the subset of samples with RNA-seq data that were in the TCGA Affymetrix testing partition (*n* = 91). All RNA-seq expression vectors were log transformed and then scaled to the range [0,1].

### Implementation details

The creation of the correlation and binary matrices as well as the expression scaling and prediction model creation and testing was performed using R 3.0.1 [34]. The following R packages were used throughout our work-flow: curatedOvarianData [27], randomForest [33], boot [35], igraph [36], doppelgangR (https://github.com/lwaldron/doppelgangR), and ggplot2 [37]. GGS was implemented using Python 2.7 (using docopt, numpy, itertools, and collections) and the entire work-flow (including the preprocessing, parameter sweep, and expression prediction) is made available [20] on github (https://github.com/greenelab/greedy-geneset-selection).

### Availability of supporting data

While for convenience, we accessed most of our data through the R package “curatedOvarianData” [27], all datasets used (other than the TCGA) are available from the Gene Expression Omnibus (GEO). Specifically: Yoshihara et al. (GEO Accession GSE32062), Tothill et al. (GEO Accession GSE9891), Bonome et al. (GEO Accession GSE26715), and Mayo (GEO Accession GSE74357). Additionally, all the code used to perform our analyses as well as the results for the TCGA breast cancer analysis are publicly available on github (https://github.com/greenelab/greedy-geneset-selection) and has a DOI assigned via zenodo: 10.5281/zenodo.32087.

## Additional files

Additional file 1: Table S1. Distribution of eligible genes across PANTHER Go-slim pathways. (XLSX 22 kb) Additional file 2: Figure S1-S6. Additional figures provided illustrate network structure of GGS selected and predictable genes, parameter sweep results using candidate genesets, imputation accuracy using candidate genesets, and imputation accuracy in RNA-seq data. (PDF 1622 kb) Additional file 3: Table S2. Summary of GGS parameter sweep and expression prediction results. (XLSX 46 kb) Additional file 4: Table S3. Filtering of samples using inclusion criteria. (XLSX 9 kb)

## Abbreviations

DM: directly measured gene set (i.e., the genes selected to be assayed)
GGS: greedy gene set selection algorithm
HGSC: high grade serous ovarian cancer
r_P_: pearson correlation coefficient
r_S_: spearman rank correlation coefficient
TCGA: The Cancer Genome Atlas
