# Supplementary material for: Leveraging global gene expression patterns to predict expression of unmeasured genes
Source: BMC Genomics. 2015 Dec 15;16:1065. doi: 10.1186/s12864-015-2250-5 (PMC4678722; doi:10.1186/s12864-015-2250-5)
Supplement: Additional file 2: Figure S1-S6. — Additional figures provided illustrate network structure of GGS selected and predictable genes, parameter sweep results using candidate genesets, imputation accuracy using candidate genesets, and imputation accuracy in RNA-seq data. (PDF 1622 kb) [file 12864_2015_2250_MOESM2_ESM.pdf]

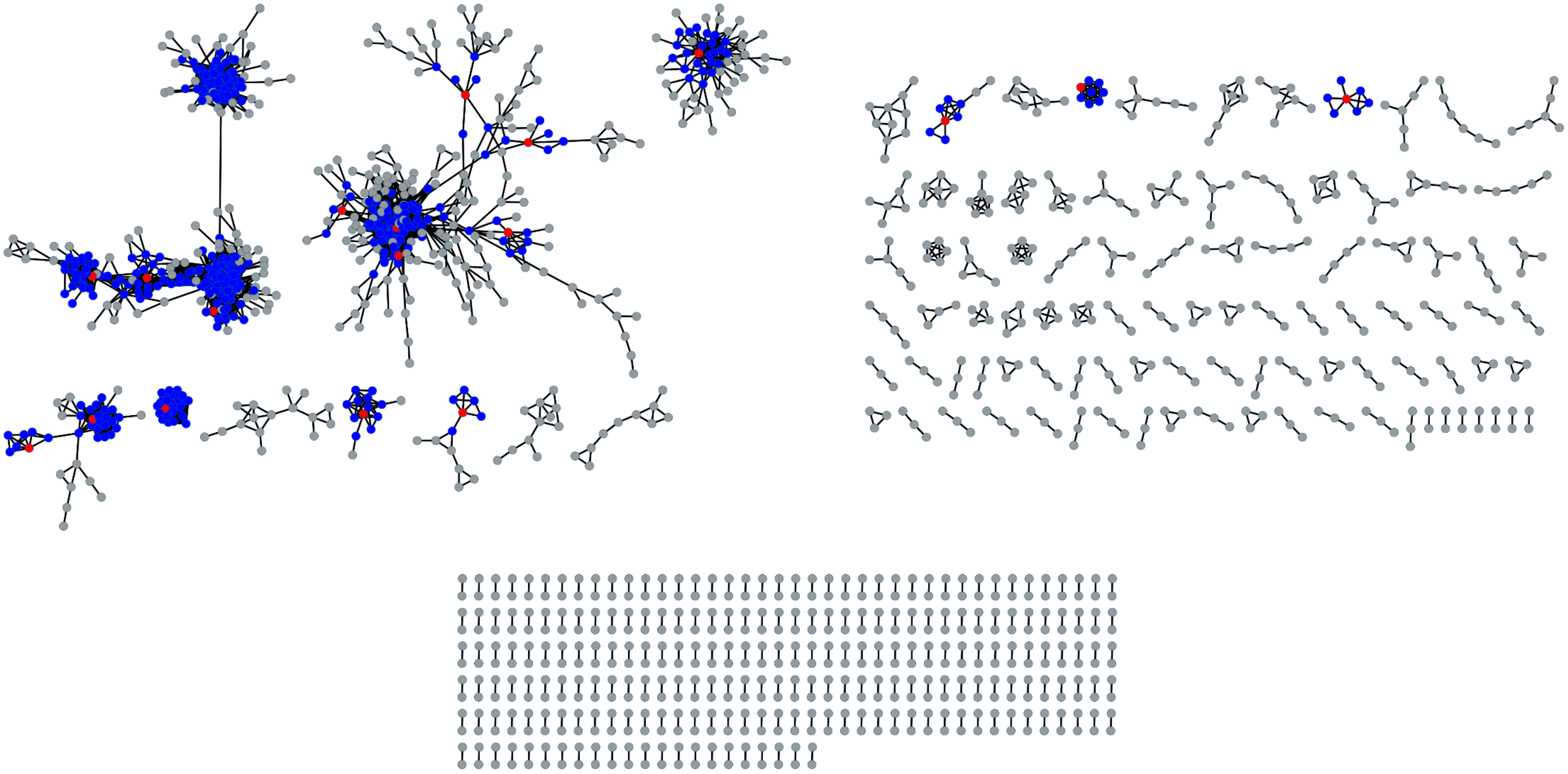

Figure S.1: Network of eligible genes with 20 directly measured genes and associated predictable genes. Network representation of the 1,577 genes connected to at least one other gene at the $|r_P|$ threshold of 0.70, in TCGA data. Genes are nodes, and undirected edges exist if the genes are correlated above the 0.70 threshold. Based on GGS with $|r_P|$ of 0.70, DM size of 20, and redundancy of 1, the 20 DM genes are depicted in red, the 430 predictable genes are blue, and the 1,127 genes that are eligible but were not selected for either the DM or predictable sets are gray.

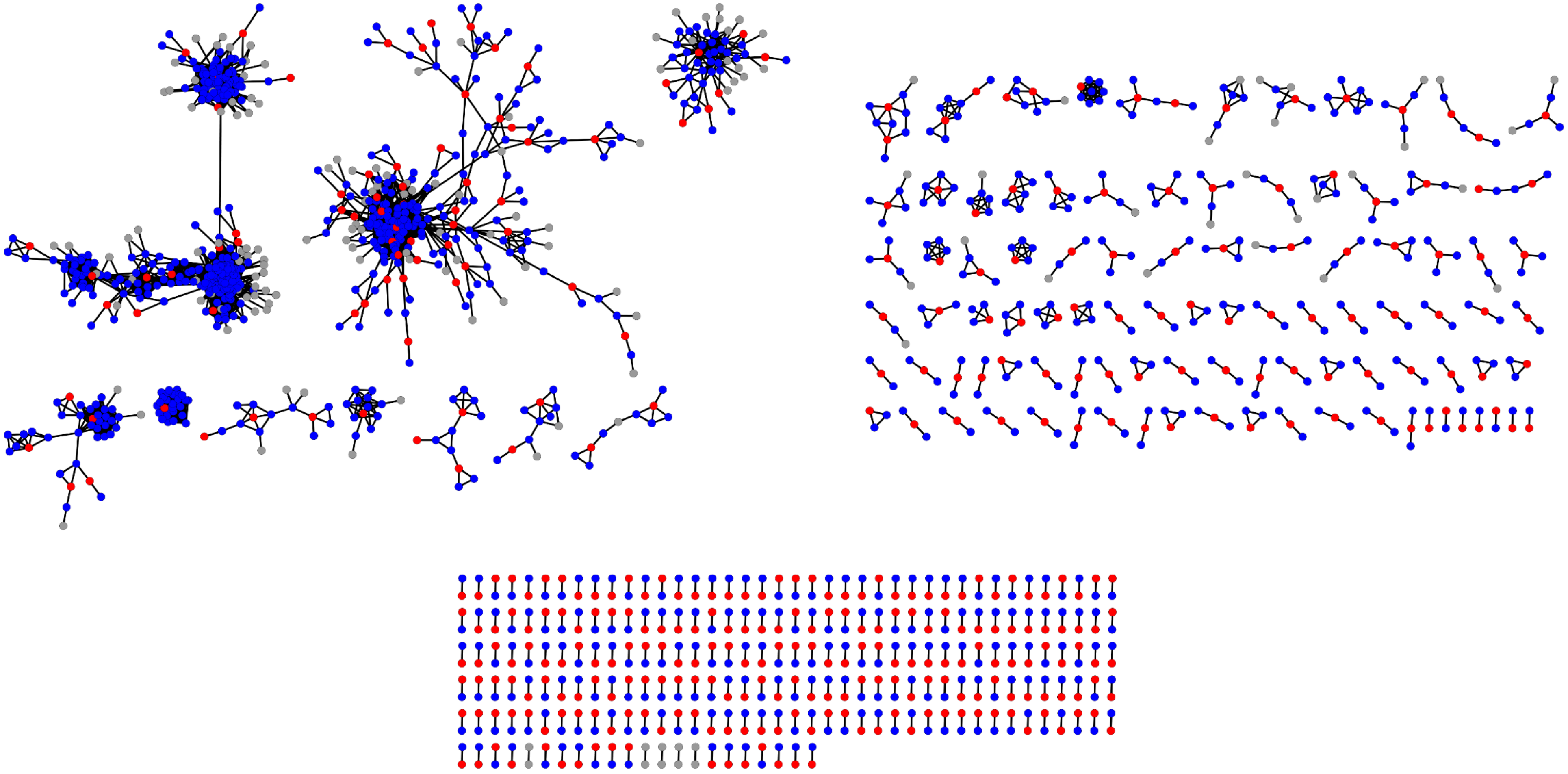

Figure S.2: Network of eligible genes with 400 directly measured genes and associated predictable genes. Network representation of the 1,577 genes connected to at least one other gene at the |rP| threshold of 0.70, in TCGA data. Genes are nodes, and undirected edges exist if the genes are correlated above the 0.70 threshold. Based on GGS with |rP| of 0.70, DM size of 400, and redundancy of 1, the 400 DM genes are depicted in red, the 1,018 predictable genes are blue, and the 159 genes that are eligible but were not selected for either the DM or predictable sets are gray

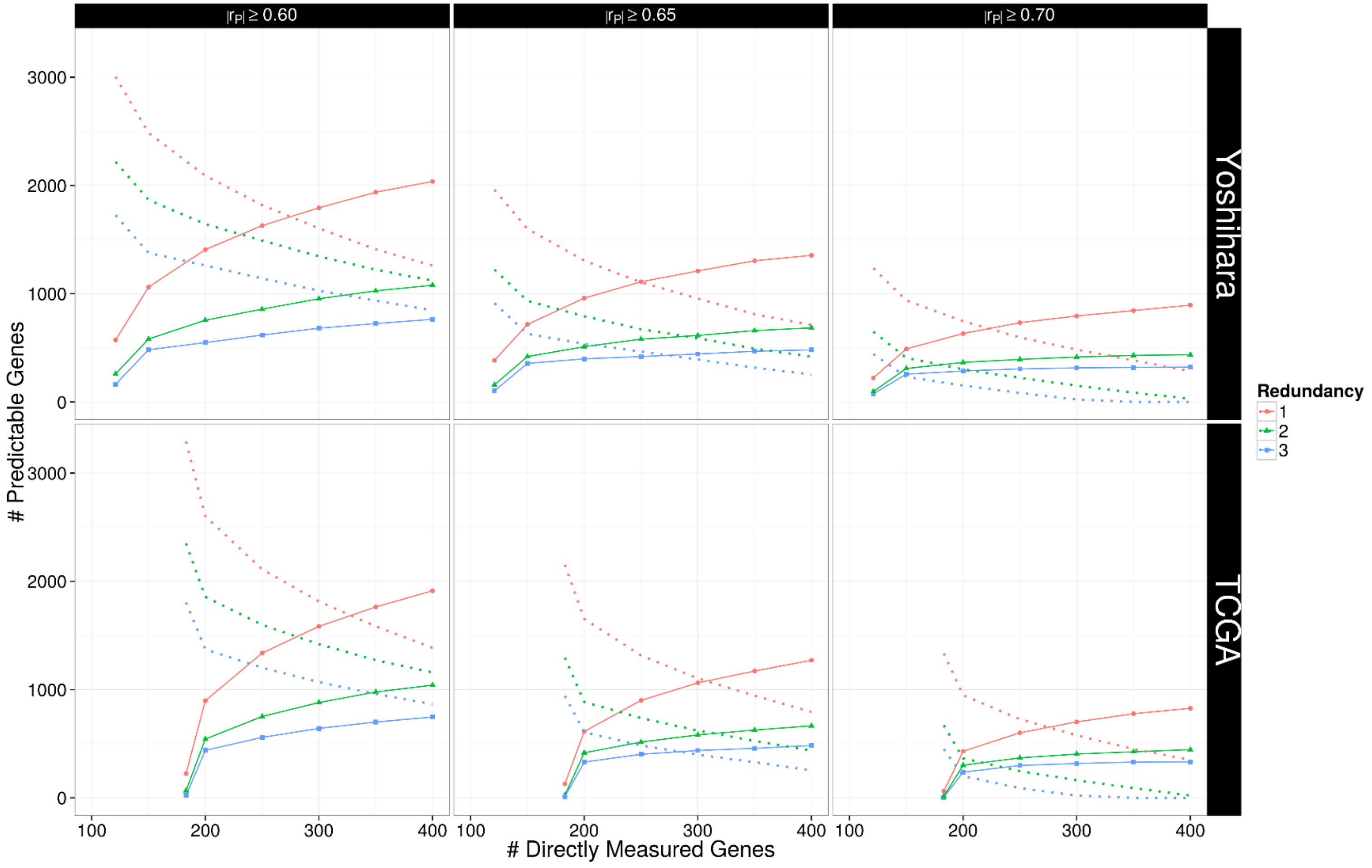

Figure S.3: Predictable geneset size across GGS parameters for Yoshihara et al. and TCGA candidate genesets. The number of predictable genes by fold redundancy (1-red circle; 2-green triangle; 3-blue squares), number of directly measured genes (x-axis), and correlation threshold (column facets). Solid lines indicate the number of predictable genes given a GGS selected directly measured geneset of size indicated by the x-axis. The dotted line indicates the remaining eligible genes that are neither predictable nor directly measured. Results using the Yoshihara candidate genes are in the upper row, and results using the TCGA candidate genes are in the lower row.

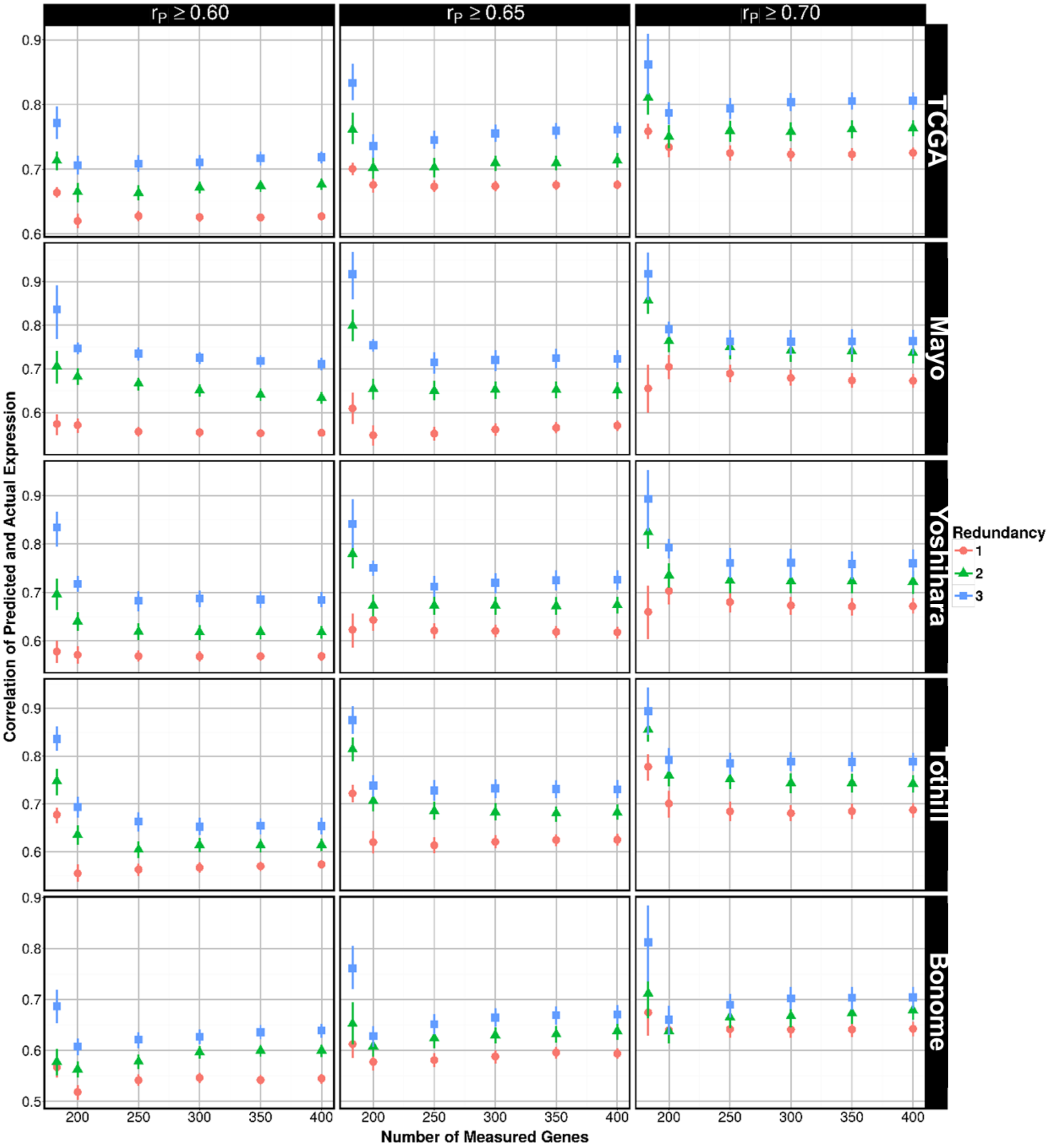

Figure S.4: Expression imputation accuracy using the TCGA candidate geneset. Average expression prediction accuracy by number of directly measured genes including the TCGA candidate gene set (x-axis), fold redundancy (color), correlation threshold (columns), and dataset (rows). DM sets indicated by the x-axis include the 183 TCGA candidate genes. The y-axis indicates the mean and bootstrapped standard error of the Spearman rank correlation (r_s) between actual expression and predicted expression.

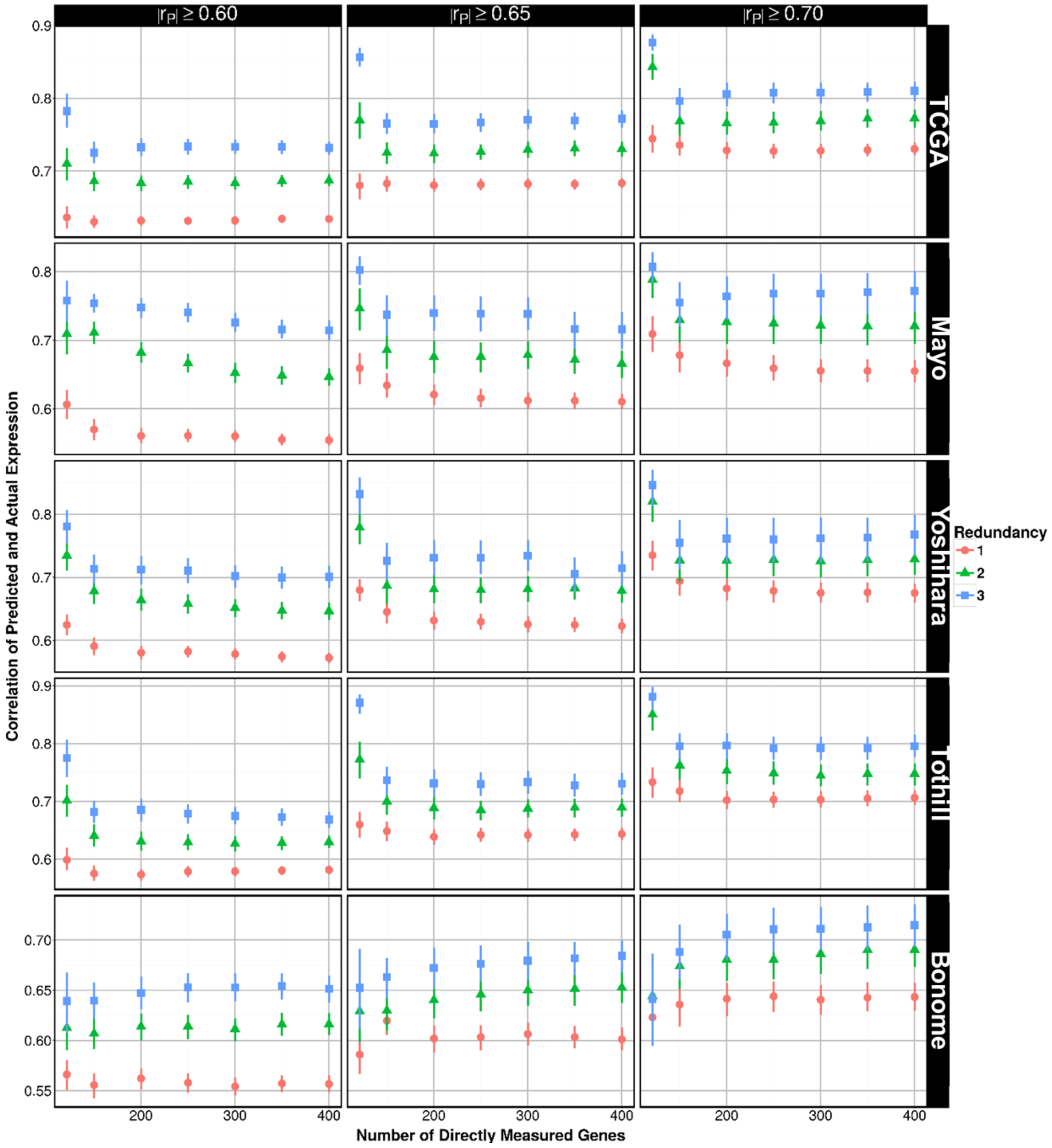

Figure S.5: Expression imputation accuracy using the Yoshihara et al. candidate geneset. Average expression prediction accuracy by number of directly measured genes including the Yoshihara candidate gene set (x-axis), fold redundancy (color), correlation threshold (columns), and dataset (rows). DM sets indicated by the x-axis include the 121 Yoshihara candidate genes. The y-axis indicates the mean and bootstrapped standard error of the Spearman rank correlation (r_s) between actual expression and predicted expression.

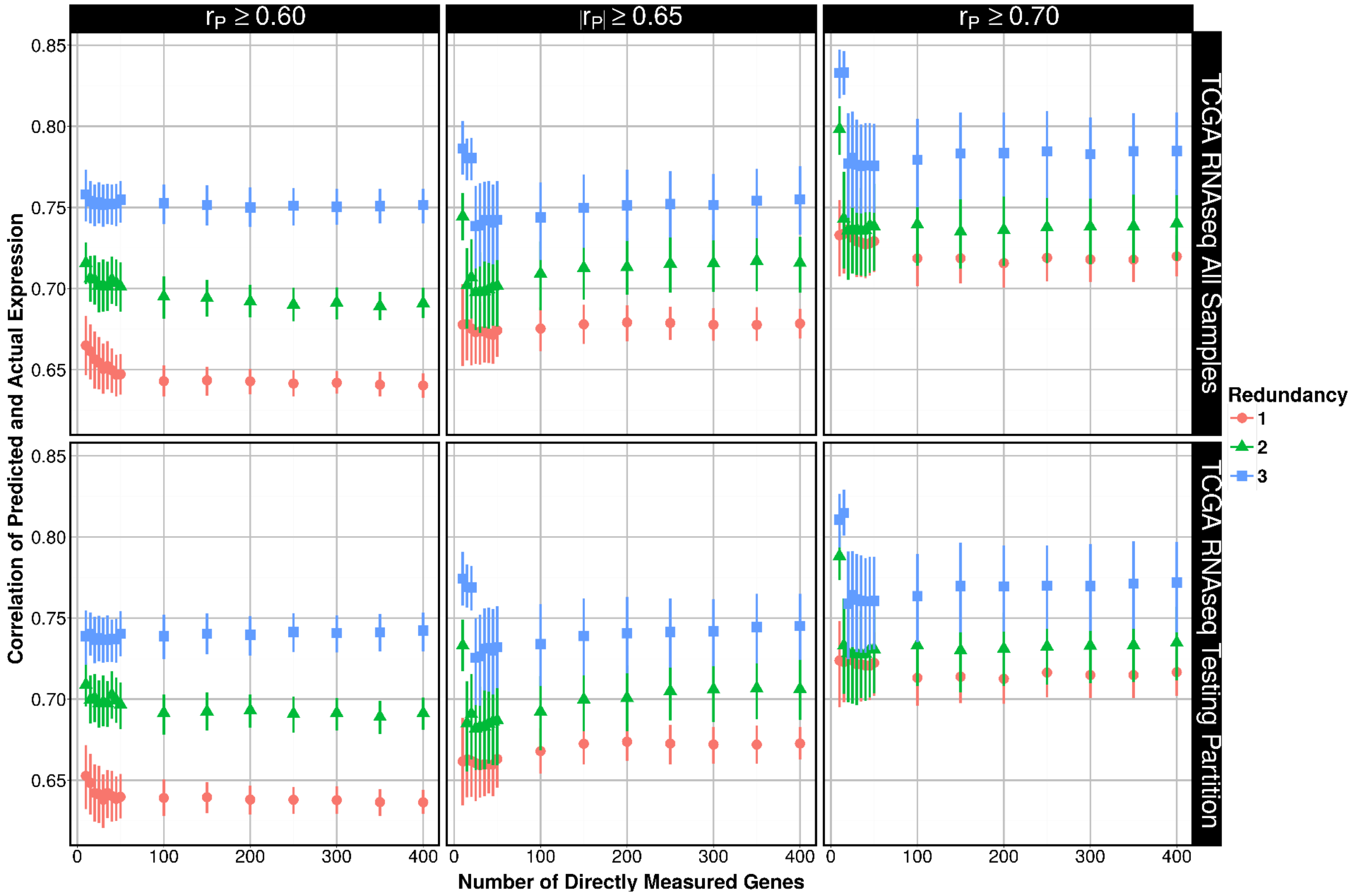

Figure S.6: Expression imputation accuracy in TCGA RNA-seq data. Average expression prediction accuracy in RNA-seq data by number of directly measured genes (x-axis), fold redundancy (color), and correlation threshold (columns). The top row includes all 236 samples that had both array and RNA-seq expression data. The bottom row includes only the subset of RNA-seq samples whose array data were used as testing data. The y-axis indicates the mean and bootstrapped standard error of the Spearman rank correlation (rs) between actual expression and predicted expression.
